# 1797. Evaluation of Intravenous vs Oral Antibiotic Therapy at Discharge for the Treatment of Urinary Source Gram-Negative Bacteremia

**DOI:** 10.1093/ofid/ofac492.1427

**Published:** 2022-12-15

**Authors:** Xuping Yan, Matthew Song, Sarah E Moore, Elena A Swingler, Ashley M Wilde, Brian C Bohn

**Affiliations:** Norton Healthcare, Louisville, Kentucky; Norton Healthcare, Louisville, Kentucky; Norton Healthcare, Louisville, Kentucky; Norton Healthcare, Louisville, Kentucky; Norton Healthcare, Louisville, Kentucky; Barnes-Jewish Hospital, St. Louis, Missouri

## Abstract

**Background:**

Retrospective studies demonstrate similar efficacy between oral (PO) and intravenous (IV) antibiotics at discharge for gram negative bacteremia (GNB), with reduced duration of hospitalization, hospital costs, and duration of therapy with PO. Understanding current physician discharge practices can help guide future antimicrobial stewardship initiatives.

**Methods:**

A multicenter, retrospective cohort study was conducted on adult inpatients admitted between July 1, 2020 and June 30, 2021 with urinary source GNB. Patients with both blood and urine cultures positive for *Escherichia coli, Klebsiella* species, or *Proteus mirabilis* and a PO antibiotic available at discharge were included. The primary outcome was the percentage of patients discharged on PO antibiotics. Secondary outcomes included duration of hospitalization, total length of therapy, and 30-day readmission secondary to clinical failure or therapeutic complication in the IV and PO at discharge groups.

**Results:**

Of the 157 included patients, 128 (82%) were discharged on PO antibiotics. Patients discharged on IV and PO had similar median quick Pitt bacteremia scores [1(IQR, 0 – 2) vs. 1 (IQR, 0 – 1), p=0.420]. Mean duration of hospitalization for patients discharged on IV vs. PO therapy was 5 (SD, 2.91) days vs. 4 (SD, 2.75) days, p=0.001, respectively. Mean total length of antibiotic therapy was 21.6 (SD, 13.80) days as compared to 13.5 (SD, 4.94) days, p=0.017, for the IV vs. PO arms, respectively. Thirty–day readmission rates were 11/29 (38%) and 23/128 (18%), p=0.035, in the IV and PO arms, respectively. Notably, 21/29 (72%) of the IV arm had an organism from blood culture that was susceptible to trimethoprim-sulfamethoxazole (TMP-SMX).

**Figure:**
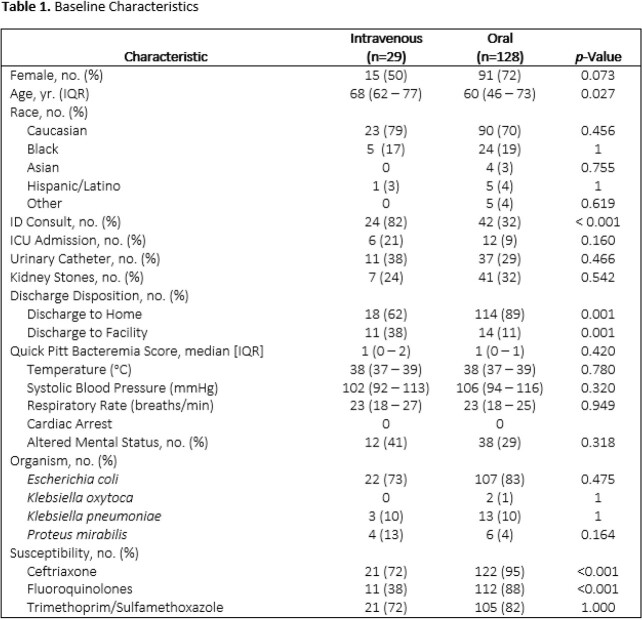

**Figure:**
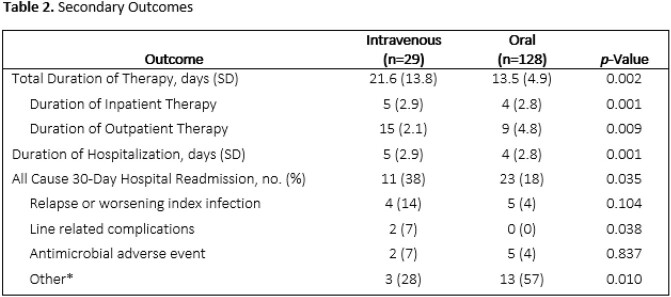

**Conclusion:**

Most patients with urinary source GNB were discharged on PO antibiotics. Patients discharged on IV antibiotics were more likely to be readmitted within 30 days. Future efforts to decrease IV antibiotic use may focus on increasing TMP-SMX use at discharge.

**Disclosures:**

**Matthew Song, PharmD, BCIDP**, Merck: Stocks/Bonds|ObsEva: Stocks/Bonds.

